# A high-throughput phenotyping dataset for GWAS analysis of maize under combined drought and heat stress

**DOI:** 10.1016/j.dib.2025.111947

**Published:** 2025-08-05

**Authors:** Rongli Shi, Ana López-Malvar, Dominic Knoch, Henning Tschiersch, Marc C. Heuermann, Salar Shaaf, Delphine Madur, Rogelio Santiago, Carlotta Balconi, Elisabetta Frascaroli, Sekip Erdal, Carine Palaffre, Alain Charcosset, Pedro Revilla, Thomas Altmann

**Affiliations:** a Leibniz Institute of Plant Genetics and Crop Plant Research (IPK), OT Gatersleben, 06466 Seeland, Germany; b Universidad de Vigo, As Lagoas Marcosende, Agrobiología Ambiental, Calidad de Suelos Y Plantas (UVIGO), Unidad Asociada a La MBG (CSIC), 36310 Vigo, Spain; c Université Paris-Saclay, INRAE, CNRS, AgroParisTech, Génétique Quantitative et Evolution (GQE) - Le Moulon, 91190 Gif-Sur-Yvette, France; d Misión Biológica de Galicia (CSIC), Carballeira 8, 36143 Pontevedra, Spain; e Research Centre for Cereal and Industrial Crops (CREA), Via Stezzano, 24, 24126 Bergamo, Italy; f Department of Agricultural and Food Sciences, Università di Bologna, Viale Fanin, 44 40127 Bologna, Italy; g Bati Akdeniz Agricultural Research Institute, Antalya, Türkiye; h Unité Expérimentale du Maïs, INRAE, 2297 Route de l’INRA, F-40390 Saint-Martin-de-Hinx, France

**Keywords:** Drought, Heat, High-throughput phenotyping, Mediterranean germplasm, Photosynthesis, Vegetative growth

## Abstract

This dataset was generated to characterize the physiological and morphological mechanisms underlying tolerance and resilience to combined drought and heat stress using a panel of 106 Mediterranean maize inbred lines. To achieve this, high-throughput non-invasive phenotyping combined with genome-wide association analysis was applied to accurately capture the dynamic responses of the maize lines to stress and to dissect the genetic basis of maize tolerance and resilience. Two experiments were conducted under control (25/20 °C, 70 % field capacity (FC)) and stress conditions (35/25 °C, 30 % FC). Stress was applied from 18 to 32 DAS (days after sowing), followed by a recovery period under control conditions. Plants were grown under controlled air temperature and soil water content, and were harvested at 45 DAS. Throughout the cultivation period, multiple camera sensors captured images daily, allowing agronomic traits to be extracted for analysis. The dataset includes raw and processed images, phenotypic data obtained from these images, results of two photosynthesis related parameters, Genome-Wide Association Study (GWAS) results from one parameter as an example, and scripts used for data analysis. Additionally, metadata and a detailed description of the experimental setup are provided. This resource is suitable for researchers interested in stress phenotyping and quantitative genetics. It allows further exploration of genotype-by-environment interactions and integration with other omics datasets. The dataset provides a valuable foundation for studies aiming to understand and improve crop resilience to climate-related abiotic stresses.

Specifications Table

**Table:** 

Subject	Biology
Specific subject area	Morphological and physiological responses of maize to abiotic stress, Genome-Wide Association Study (GWAS)
Type of data	This dataset is organized into three main directories: Raw and analysed images, processed data and metadata (MIAPPE).
Data collection	Data were collected using a high-throughput shoot phenotyping platform in an environmentally controlled greenhouse, assessing 106 maize lines during vegetative growth under two conditions: control (non-stress) and combined drought and heat stress. These lines are sourced from Mediterranean regions or adapted to Mediterranean environments.
Data source location	Leibniz Institute of Plant Genetics and Crop Plant Research; Corrensstrasse 3, D-06466 Seeland, OT Gatersleben, Germany)
Data accessibility	The produced raw datasets and source code were uploaded to the e!DAL repository in ISA-Tab format (http://dx.doi.org/10.5447/ipk/2025/8) according to the MIAPPE standard.
Related research article	Shi, R., López-Malvar, A., Knoch, D., Tschiersch, H., Heuermann, M. C., Shaaf, S., Madur, D., Santiago, R., Balconi, C., Frascaroli, E., Erdal, S., Palaffre, C., Charcosset, A., Revilla, P., & Altmann, T. (2025) Integrating high-throughput phenotyping and genome-wide association analyses to unravel Mediterranean maize resilience to combined drought and high temperatures. **Plant Stress** DOI: 10.1016/j.stress.2025.100954

## 1 Value of the Data

• This phenotypic dataset allowed for detailed, dynamic analysis of vegetative maize responses—both morphological and physiological—to concurrent drought and heat stress. Multiple abiotic stresses can be assessed using photosynthetic parameters as indicators. The assessed stress adaptability of the tested lines supports their use in breeding programs focused on enhancing tolerance to multiple abiotic stresses.
• These data contribute to the understanding of the genetic basis of complex traits, identifying functional genes linked to stress resilience, ultimately supporting the development of improved, stress-tolerant varieties for more sustainable agriculture.
• This dataset can assist other researchers in developing their phenotyping pipelines and can be reused to estimate plant responses to multiple abiotic stresses or to model and simulate improvements in agriculture.

## 2 Background

Due to climate changes, plants are more frequently exposed to multiple stress factors, such as drought and heat waves. High-throughput imaging-based phenotyping has been widely employed in the recent decade since it is an automated non-invasive precise and fast technique. It presents huge potential in the context of understanding the morphological, physiological and genetics of stress resilience [1] Kinetic chlorophyll fluorescence measurements in high-throughput phenotyping platforms enable the assessment of photosynthetic activity, a critical factor in plant stress resistance [2]. On the other side, integration of high-throughput phenotyping and genome-wide association studies (GWAS) offers an efficient and powerful approach to dissect the genetic basis of abiotic stress underlying complex agronomic traits [3,4]. Maize is one of the most important staple crops worldwide, and there is an urgent need to develop genotypes that are resilient to multiple abiotic stresses. We conducted two experiments and generated phenotypic data to gain deeper insights into the underlying mechanisms of maize responses to combined drought and heat stress, to investigate the genetic architecture of complex traits, and to support the development of climate-resilient maize genotypes.

## 3 Data Description

This dataset is stored in the e!DAL repository in ISA-Tab format (http://dx.doi.org/10.5447/ipk/2025/8) according to the MIAPPE standard. It consists three main folders: raw data; processed data and R code; and MIAPPE. A simple description for the experiments is in Abstract.txt and the main contents are in README.txt.

Below is a detailed overview of the folder structure and contents (Table 1).

**Table 1:** Available data information.

Path	File Name	Content Description
/raw data	2228AL 2237RS	Both of these two folders contain the output of the IAP analysis of the respective experiment: the raw images (data/Maize), the processed images (data/Analysis Results), the pre-view images (icons), the conditions files (conditions), the tuned IAP pipeline (*pipeline.ini), the obtained report file (_report.csv).
	2228AL_watering_Stress.csv 2237RS_watering_Control.csv	Watering record for both experiments.
	Metadata_Control.csv Metadata_Stress.csv	The lines/genotypes correspond to the carrier IDs in the system for both experiments.
	photosynthesis traits_Control.xlsx photosynthesis traits_Stress.xlsx	The photosynthesis related two parameters obtained from the two experiments.
	Genodata_SNPs.csv	The genetic data of 100 lines.
/ processed data and R code:	input output	The dataset used for the R codes are in folder “input” and the outcomes are in folder ``output''.
	repeatability and BLUPs_Control.R repeatability and BLUPs_Stress.R	R code for calculating repeatability and BLUPs for both experiments
	GWAS_GAPIT.R	R code for GWAS analysis by using GAPIT package. The phenotype lab_a is given as one example.
	BLUPs_treatment_106.csv	Final BLUPs used for further analysis or visualization for both experiments.
	Repeatability figures.xlsx	Figures for repeatability were done in excel after calculated from models in R code.
	Stress indices.xlsx	The stress indices were calculated in this excel.
/MIAPPE	a_2228AL_phenotyping.txt a_2237RS_phenotyping.txt i_investigation_DROMAMED_MAIZE.txt s_2228AL_study.txt s_2237RS_study.txt	It contains the investigation file, study file and assay file in ISA-Tab format meeting the MIAPPE standard

## 4 Experimental Design, Materials and Methods

### 4.1 Plant materials and growth condition

The plant material consisted of 106 maize inbred lines, representative of Mediterranean regions or adapted to Mediterranean environments. These lines were sourced from gene banks in Spain (44), Italy (27), Turkey (13) and France (22), and may serve as valuable sources of alleles that enhance tolerance to drought and/or high temperature stress. They have never been evaluated together under the same growth conditions.

The phenotypic data were collected from the automated high-throughput shoot phenotyping platform at the Leibniz Institute of Plant Genetics and Crop Plant Research (IPK), Gatersleben, Germany [5].

The experimental activity involved two parts: one under control (non-stress, 2237RS) conditions and the other under stress (combined drought and heat conditions, 2228AL). Under non-stress conditions, the environment was maintained at 25 °C air temperature during the day and 20 °C at night (16/8 h photoperiod), with 60 % relative air humidity and soil moisture held at 70 % of field capacity (FC). Under stress conditions, plans were exposed to air temperatures of 35 °C day / 25 °C night (16/8 h day/night), with 40 % relative air humidity and a soil moisture of 30 % FC.

**Fig. 1:**
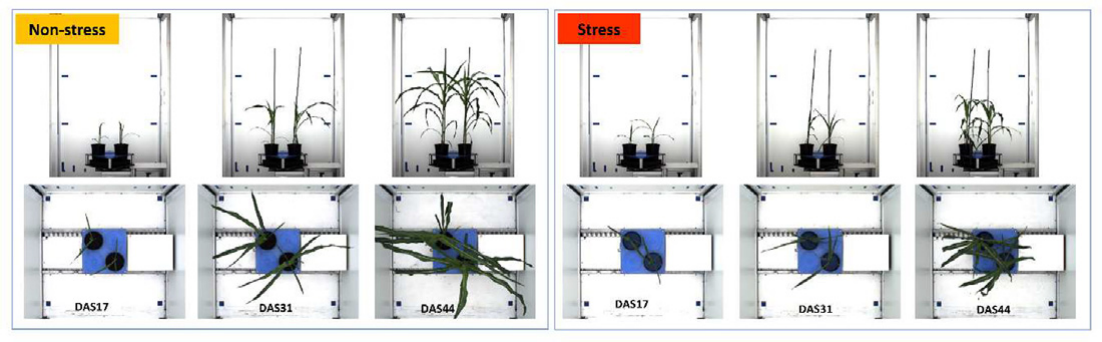
Example side and top view images of plants grown in individual pots and arranged as two plants per carrier taken in the RGB (VIS) imaging chamber of IPKs automated plant phenotyping platform for large plants. Plants of the maize inbred line B73 imaged at three time points during growth under non-stress (left) and stress conditions (right), respectively, are shown as representative examples. DAS: days after sowing. Stress period was between 18-32 DAS.

For both experiments, seedlings of plants were transferred to the phenotyping system 10 days after sowing (DAS). The experimental design was a randomized block with six replicates, employing three carriers per line, each holding two pots with one plant per pot. To prevent excessive overlapping of plant canopies, the two pots within each carrier were arranged diagonally (Fig. 1). The system comprises a total of 12 lanes, grouped into three blocks of four lanes each. The three replicate carriers for each line were distributed across the three blocks to ensure randomized and balanced representation. Watering was controlled by weighing using targeted weights and kept at 70 % FC or 30 % FC daily, for non-stress and stress experiments, respectively. Stress conditions were imposed at 18 DAS and maintained for two weeks. Before and after the stress period, plants were grown under control conditions. In both experiments, plants were cultivated until 45 DAS. The amount of water supplied to each carrier in both experiments was recorded and is included in the dataset.

### 4.2 Phenotypic assessment and data analysis

Top and side views of RGB images of the tested lines were captured six times per week during the control experiment and five times per week during the stress experiment throughout the growth period. On the remaining days of the week, kinetic chlorophyll fluorescence image analysis (FluorCam) was performed for each carrier (two pots/carrier). PSII operating efficiency (ΦPSII) was measured during the day using high-throughput-optimized protocols according to Tschiersch et al. [2]. The plants were light acclimated in the plant adaptation tunnel for at least 5 min followed by 10 s illumination at the same light intensity (PAR 350 µmol photons *m*^−2^ s^−1^) after moving into the kinetic chlorophyll fluorescence imaging chamber. Finally, maximum Fm′was measured during 800 ms exposure to a saturating light flash (PAR: 4100 µmol photons *m*^−2^ s^−1^). Maximum quantum efficiency of PSII (Fv/Fm) was measured at night (2 h after onset of the dark period). In the dark-adapted state minimal fluorescence level (F0) was determined by the application of a weak, pulsed measuring light (PAR ≤ 0.2 µmol photons *m*^−2^ s^−1^) that does not drive photosynthesis and a saturating light pulse (800msec; PAR: 4100 µmol photons *m*^−2^ s^−1^) was applied to induce transiently maximal fluorescence level (Fm). Variable fluorescence (Fv) was calculated as: Fv = Fm –F0 and maximum quantum yield of PSII was given as Fv/Fm. These protocols were designed to be compatible with our high-throughput facility, which has a maximum capacity of 45 carriers/h for ΦPSII and 63 carriers/h for Fv/Fm measurement. During image analysis process, the two plants in each carrier were separated, and individual values were extracted per plant.

The obtained RGB and FLUOR images were analyzed using the IAP (Integrated Analysis Platform) software [6]. The basic maize pipeline was initially used and then adjusted based on the pre-processed images. The adapted pipeline was subsequently applied for further analysis. For each experiment, >500 phenotypic traits were extracted per carrier at each time point. The analysis was performed on all carriers. Carriers with dead plants or one plant that was extremely small were excluded from further analysis. Descriptions of selected trait parameters can be found in Shi et al. [7].). For instance, estimated shoot volume (ESV) was calculated based on both top and side visible RGB information. The volume was computed using the following formula: Volume = Math.sqrt (t1 * s2 * s3 / 2d * Math.sqrt (1 - Math.pow ((s2 * s2 + s3 * s3 - s1 * s1) / (2d * s2 * s3), 2))). Where t1 denotes avgTopArea; s1 denotes sideArea_for_angleNearestTo0; s2 denotes sideArea_for_angleNearestTo45; s3 denotes sideArea_for_angleNearestTo90, d is a constant (here is 1) [6].

**Fig. 2:**
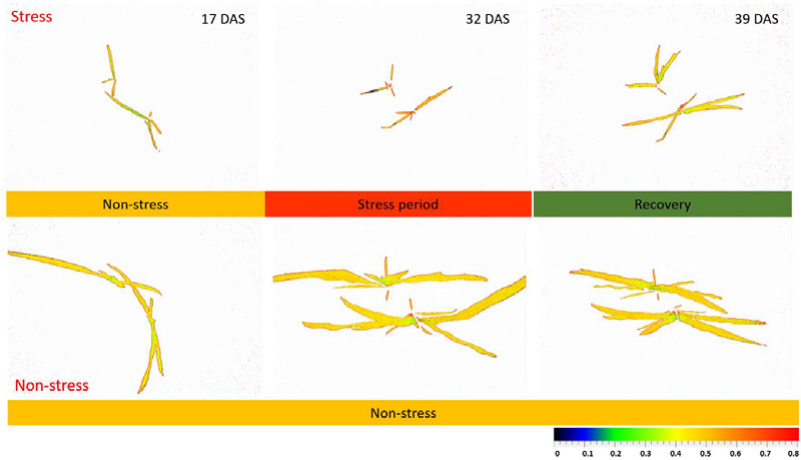
PSII operating efficiency of line EP2020–26 over time under both experimental conditions. DAS: days after sowing, Non-stress: control experiment, Stress: combined drought and heat stress.

Fluorescence images were captured from the top view of plants using the integrated FluorCam device (Photon Systems Instruments, Czech Republic). The derived images were processed using automated image analysis routines which are implemented in the FluorCam software package according to Tschiersch et al. [3] and Lauterberg et al. [8]. Two parameters related to photosynthesis, PSII operating efficiency (ΦPSII) and maximum quantum efficiency of PSII (Fv/Fm) were obtained for each plant. There were four and seven measurement time points of these two traits through the growth period for the non-stress and stress experiments, respectively. One example is shown in Fig. 2. To minimize environmental effects, repeatability and BLUPs of all phenotypic data were calculated using R software and used for visualization and further comparation between the treatments.

### 4.3 Genotyping and genome-wide association study (GWAS) analysis

DNA was extracted following Tai and Tanksley [9] with minor modifications by using leaf samples from 100 inbred lines at 2–3 leaf stage. Genotyping was performed using the Axiom™ Maize 600k Genotyping Array according to the manufacturer’s instructions [10]. Data were analyzed with the dedicated Axiom Analysis Suite software (ThermoFisher Scientific) v5.1.1.1 using r4 library files. The obtained 616,201 SNPs were filtered (MAF ≥ 0.05 and missing rate ≤ 10 %) and missing SNP calls were imputed using the BEAGLE v.5.2 [11]. After imputation, LD pruning of SNPs was performed by using the R package SNPRelate, [12]. In total, 191,205 SNPs were used for GWAS analysis, which was performed using the GAPIT package in R [13].

## Limitations

‘Not applicable’.

## Ethics Statement

Authors have read and follow the ethical requirements for publication in Data in Brief and confirming that the current work does not involve human subjects, animal experiments, or any data collected from social media platforms.

## CRediT authorship contribution statement

**Rongli Shi:** Methodology, Investigation, Data curation, Writing – original draft. **Ana López-Malvar:** Methodology, Investigation, Data curation, Writing – original draft. **Dominic Knoch:** Data curation, Visualization, Writing – review & editing. **Henning Tschiersch:** Methodology, Writing – review & editing. **Marc C. Heuermann:** Data curation, Visualization, Writing – review & editing. **Salar Shaaf:** Data curation, Visualization, Writing – review & editing. **Delphine Madur:** Methodology. **Rogelio Santiago:** Resources, Writing – review & editing. **Carlotta Balconi:** Resources, Writing – review & editing. **Elisabetta Frascaroli:** Resources, Writing – review & editing. **Sekip Erdal:** Resources, Writing – review & editing. **Carine Palaffre:** Resources, Writing – review & editing. **Alain Charcosset:** Resources, Writing – review & editing. **Pedro Revilla:** Resources, Writing – review & editing. **Thomas Altmann:** Funding acquisition, Conceptualization, Writing – review & editing.

## Data Availability

CCDCHigh-throughput shoot phenotyping of maize under combined drought and heat stress (Original data).
